# Evidence-Based Interventions for High Blood Pressure and Glycemic Control Among Illinois Health Systems

**DOI:** 10.5888/pcd17.190058

**Published:** 2020-01-23

**Authors:** Janae D. Price, Manasi Jayaprakash, Cristina M. McKay, Nancy L. Amerson, Paula L. Jimenez, Kamil E. Barbour, Timothy J. Cunningham

**Affiliations:** 1 Division of Population Health, National Center for Chronic Disease Prevention and Health Promotion, Centers for Disease Control and Prevention, Atlanta, Georgia; 2 Illinois Department of Public Health, Office of Health Promotion, Division of Chronic Disease Prevention and Control, Springfield, Illinois; 3 Illinois Primary Health Care Association, Clinical Services and Workforce Development, Division of Clinical Services, Springfield, Illinois

## Abstract

**Introduction:**

Evidence-based interventions (referral, team-based care, self-management, and self-monitoring) for chronic disease management are well documented and widely used by Federally Qualified Health Centers (FQHCs). However, how these interventions are implemented varies substantially.

**Methods:**

The Illinois Health Information Systems Survey was deployed to 49 FQHCs. Responses were grouped into 4 distinct policies, systems, and processes (P/S/P) categories: internal policies/workflows, huddles (brief meetings), electronic health record alerts/tracking tools, and case manager/coordinator interaction. Responses were then direct-matched to the 2016 Health Resources and Services and Administration Uniform Data System clinical quality indicator (QI) percent scores. Descriptive statistics were generated and level of significance (*P* < .05) was tested for hypertension and type 2 diabetes mellitus.

**Results:**

The total number of P/S/Ps in place for hypertension ranged from 0 to 13 (mean, 6.9) and 0 to 8 for diabetes (mean, 5.1). Meeting or exceeding the national mean QI percent score for controlled blood pressure (62.4%) was significant among FQHCs with 9 or more P/S/Ps compared with those with 8 or fewer P/S/Ps. A positive association in clinical QI percent score was found among organizations that had 3 or more P/S/Ps (for all 4 intervention areas), although none were significant.

**Conclusion:**

An assessment of the types of P/S/Ps used to implement evidence-based interventions for hypertension and diabetes management is a first in Illinois. Initial results support some relationship between the number of P/S/Ps implemented and clinical QI percent score for both hypertension and diabetes.

**Summary**

**What is already known on this topic?**

Analyses of health systems interventions on chronic disease management and the variation of clinical quality improvement percent scores is limited.

**What is added by this report?**

This study underscores the variability that can occur within and across federally qualified health centers in Illinois around the number and type of policies, systems, and processes implemented.

**What are the implications for public health practice?**

When considering the tactics of implementing evidence-based chronic disease management, health systems should consider the difference between the number and types of policies, systems, and processes that address hypertension and diabetes control.

## Introduction

Hypertension and diabetes are risk factors for cardiovascular disease (CVD) and related conditions and can lead to premature death (1). Approximately 1 in 7 health care dollars is spent on CVD (2). In the United States, hypertension affects nearly 78 million adults aged 18 years or older and is a major modifiable risk factor for other CVDs and stroke. Additionally, it is estimated that 1 in 9 adults in the United States have diabetes (1). Diabetes (blood glucose) management plays a critical role in prevention of CVD (3). Long-term complications of high glucose levels and type 2 diabetes mellitus include CVD, renal failure, nerve damage, and retinal damage (4).

A comprehensive approach that incorporates evidence-based interventions is critical to address the multiple comorbidities and risk factors of CVD. Interventions adopted by health systems to address hypertension, diabetes, and other chronic diseases have shifted toward using a population health management approach (an interdisciplinary, customizable approach that allows health departments to connect practice to policy for change to happen locally [5]) for improved disease management and coordination of care. This is even more important in underserved communities, specifically among Federally Qualified Health Centers (FQHCs), given their resource-constrained environment (6) and the high-risk populations they serve.

Studies show a higher prevalence of hypertension and diabetes among populations served by FQHCs, such as minority and low-income groups (7–9). Nationally, FQHCs provide health care services to people who are geographically isolated or economically or medically vulnerable. Investments in FQHCs reduce costs for local health care systems and provide economic benefits for surrounding communities (10). FQHCs track demographic, clinical quality, and cost of care data and provide annual reports via the Uniform Data System (UDS) to the Health Resources and Services Administration (HRSA). Clinical quality improvement (QI) percent scores are national measures monitored by HRSA among FQHCs that receive federal funding and are used to monitor and improve the quality of perinatal, chronic disease, and preventive care services. These measures typically align with health care and public health measures such as Healthy People 2020. For controlled blood pressure, a higher clinical QI percent score is better (ie, more patients with controlled blood pressure) whereas for poor glycemic control a lower clinical QI percent score is better (ie, fewer patients with poor glycemic control). Given the variability of resources, organizational supports, community linkages, and information technology infrastructure, the ability of FQHCs to implement evidence-based interventions and improve QI percent scores can vary widely. The aim of this study was to understand variations in clinical QI percent scores by the types of evidence-based interventions implemented in support of hypertension and diabetes management within FQHCs in Illinois in 2016.

## Methods

### Database and survey instrument

This is a cross-sectional study using data from the 2017 Illinois Health Information Systems Survey (IL-HISS). The IL-HISS was developed as an original survey to support performance monitoring for a subset of chronic disease programs in the Illinois Department of Public Health (IDPH). In the survey, 21 questions were related to quality improvement policies, systems, and processes (P/S/Ps) that health systems have implemented in support of evidence-based interventions to manage patients with hypertension and diabetes (Appendix). The questions addressed evidence-based interventions such as electronic health record (EHR) capabilities, meaningful use, quality reporting, team-based care, referrals, self-monitoring, and self-management plans. Within each intervention, respondents were able to select 1 or more P/S/P types that have been implemented for the respective intervention. Responses were analyzed for common themes and grouped accordingly as either internal policies/workflows, huddles (10-minute or less stand-up meetings used to foster communication in a clinical setting [11]), EHR alerts/tracking tools, and case manager/coordinator interaction. Responses were then dichotomized as yes or no for each P/S/P category. P/S/P scores were then generated based on the number of P/S/Ps the organization had in place for each intervention. IL-HISS responses were direct-matched with the 2016 UDS file for Illinois to include demographic characteristics and clinical QI percent scores for controlled high blood pressure (<140/90 mm Hg) and poor glycemic control (hemoglobin A_1c_ >9%) (12).

### Study population

The IL-HISS was deployed to 49 Illinois Primary Health Care Association (IPHCA)-member FQHCs, with a 63.3% response rate (n = 32). Non-IPHCA member FQHCs were not excluded from participating in the survey. However, the survey was not directly promoted to those organizations (n < 10). To increase survey awareness and participation, a strategic survey dissemination process was created and carried out as follows: the survey was 1) co-developed and co-endorsed by IDPH and the IPHCA; 2) introduced and vetted through IPHCA’s Clinical Leadership committee; 3) pilot tested with clinical and QI managers from 3 FQHCs; and 4) deployed electronically through the organizations’ executive leadership and quality managers. All 3 pilot sites participated in the live survey following the testing process. All invited participants were given 3 weeks to respond to the survey with a single 1-week extension. Clinical directors or quality management teams were asked to report on activities occurring between January 1 and December 31, 2016. Descriptive statistics were generated as well as cumulative and within-category P/S/P scores for hypertension and diabetes.

### Statistical analysis

Patient demographic and clinical quality characteristics (perinatal health, preventive health screenings and services, and chronic disease management) among centers that responded to the IL-HISS survey (respondents) and centers that did not respond to the survey (nonrespondents) were compared using a 2-sample *t* test assuming equal variances, based on the Behrens-Fisher test, to determine if there were significant differences. Descriptive and statistical tests were conducted for IL-HISS respondents by using SAS version 9.4 (SAS Institute, Inc).

Chi-squared and Fisher exact test (for cells less than 5) were used to test significant differences in clinical QI mean scores (hypertension and diabetes) for systems with less than 3 versus 3 or more P/S/Ps cumulatively (across all intervention types) as well as within each intervention. Further analysis was conducted to understand the variation of the number of P/S/Ps overall and within a specific evidence-based intervention, looking specifically at meeting or exceeding Healthy People 2020 goals (13) and the 2016 national UDS mean clinical QI percent score (14) for controlled blood pressure (61.5% and 62.4%, respectively) and poor glycemic control (16.2% and 32.1%, respectively). Respondents were dichotomized on the basis of the total number of P/S/Ps selected overall and within intervention. Overall P/S/Ps were grouped based on being above or below the midpoint of the maximum number of possible P/S/Ps; 0 to 8 versus 9 to 16 for controlled blood pressure overall, 0 to 4 versus 5 to 8 for poor glycemic control overall, and 3 or more P/S/Ps for within-intervention analysis. For all analyses, *P* < .05 was considered significant.

## Results

Demographic and clinical characteristics for hypertension and diabetes varied slightly but were not significantly different between patients of survey respondents and nonrespondents. A higher mean difference between respondents and nonrespondents was noted for all racial/ethnic groups as well as for uninsured patients, but was not significant. In addition, the mean for the number of patients with hypertension and diabetes varied by less than 0.5%, and the centers’ clinical QI percent scores for the number of patients with controlled blood pressure and poor glycemic control varied by less than 2.5% between respondents and nonrespondents; both findings were not significant.

Survey respondents had a mean patient population of 23,784 (minimum, 2,658; maximum, 102,739). All respondents indicated having an EHR, 84.3% achieved Patient Centered Medical Home accreditation, 78% reported national quality measures for both hypertension and diabetes, and 53.1% used supplemental EHR software packages for QI interventions in 2016. Out of a maximum allowable P/S/P score of 16 for hypertension (4 P/S/P categories across 4 interventions), the mean number of P/S/Ps related to controlled blood pressure interventions was 6.9 (95% confidence interval [CI], 5.5–8.3) (Table 1). Out of a maximum allowable P/S/P score of 8 for diabetes (4 P/S/P categories across 2 interventions), the mean number of P/S/Ps related to poor glycemic control interventions was 5.1 (95% CI, 4.3–5.9). The mean UDS clinical QI percent score for controlled blood pressure was 64.9% and poor glycemic control was 33.1%, compared with the UDS mean of 62.4% and 32.1%, respectively (14). FQHCs that met or exceeded the Healthy People 2020 target of 61.5% for controlled blood pressure had an average of 7.7 P/S/Ps versus 6.1 P/S/Ps among those that did not meet the target. FQHCs that met or exceeded the Healthy People 2020 target of 16.2% for poor glycemic control had 5.0 P/S/Ps versus 4.7 among those that did not meet the target (Table 1).

**Table 1:** Policies, Systems, and Processes (P/S/Ps); Clinical Quality Improvement (QI) Percent Scores; and Healthy People 2020 Targets for Controlled Blood Pressure and Hemoglobin A_1c_ >9%, Illinois, 2016

Category	No. of P/S/Ps	Clinical QI Percent Score^a^				Mean No. of P/S/Ps^b^	
	Mean (95% CI)	Minimum	Maximum	Mean	SD	Below 2020 Target	Above 2020 Target
**Controlled blood pressure^c^**	6.9 (5.5–8.3)	49.3%	85.4%	64.9%	8.9	6.1	7.7
**Hemoglobin A_1c_ >9%** d	5.1 (4.3–5.9)	13.9%	62.0%	33.1%	11.6	5.0	4.7

a Clinical QI percent score data collected from the Health Resources and Services Administration Uniform Data System, 2016 Illinois Report (14).

b Mean number of P/S/Ps calculated and grouped as being above or below the Healthy People 2020 target for controlled high blood pressure (61.5%) (15) and for poor glycemic control (16.2%) (16).

c Controlled blood pressure = percentage of patients aged 18–85 years who had a diagnosis of hypertension and whose blood pressure was adequately controlled (<140/90 mm Hg) during the measurement period. Higher percentage score indicates positive clinical outcome.

d Hemoglobin A_1c_ >9% = percentage of patients aged 18–75 years with diabetes who had hemoglobin A_1c_ >9.0% during the measurement period. A lower percentage score indicates positive clinical outcome.

The most frequent P/S/P categories across hypertension and diabetes combined were internal programs/workflows (78.1%), huddles (65.6%), EHR alerts/tracking tools (53.1%), and case manager/coordinator interaction (40.6%) (Figure). These frequencies were consistent when looking at hypertension and diabetes alone.

**Figure:**
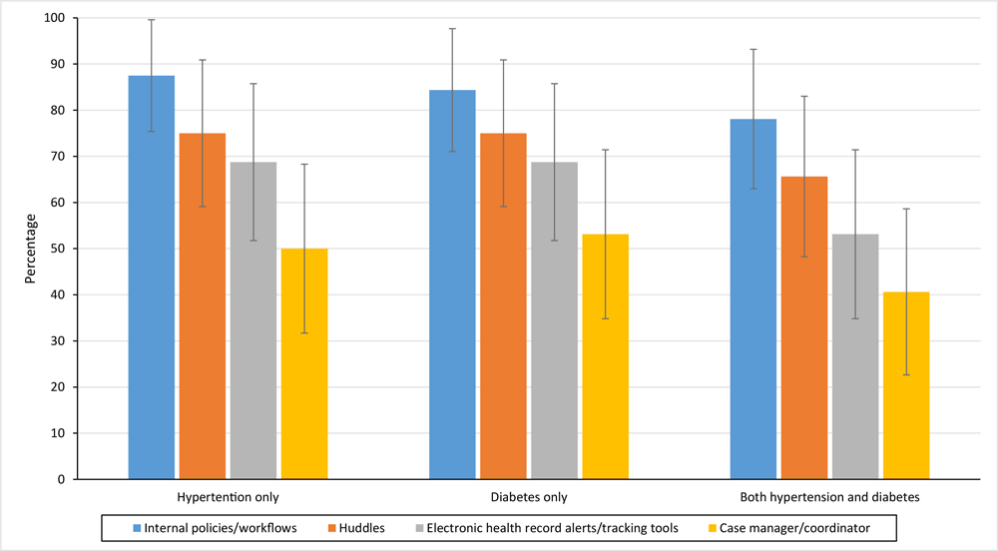
Percentage of Federally Qualified Health Centers that implemented policies, systems, and processes (P/S/Ps) for hypertension management interventions, type 2 diabetes mellitus management interventions, and both interventions combined, by P/S/P type, Illinois, 2016. Huddles are defined as 10-minute or less stand-up meetings used to foster communication in a clinical setting (11).

**Table:** 

Policies, Systems, and Processes	Hypertention Only, % (95% Confidence Interval)	Diabetes Only, % (95% Confidence Interval)	Both Hypertension and Diabetes, % (95% Confidence Interval)
Internal policies/workflows	87.5 (75.4–99.6)	84.4 (71.1–97.7)	78.1 (63.0–93.3)
Huddles	75.0 (59.1–90.9)	75.0 (59.1–90.9)	65.6 (48.2–83.0)
Electronic health record alerts/tracking tools	68.8 (51.8–85.7)	68.8 (51.8–85.7)	53.1 (34.8–71.4)
Case manager/coordinator	50.0 (31.7–68.3)	53.1 (34.8–71.4)	40.6 (22.6–58.6)

The highest percentage of P/S/Ps was the use of internal programs/workflows for the following interventions: referral to self-management programs for hypertension (81.2%; 95% CI, 66.9–95.5), referral to self-management programs for diabetes (80.6%; 95% CI, 65.9–95.4), and team-based care for diabetes (75.0%; 95% CI, 59.1–90.7) (Table 2). Huddles and EHR alerts/tracking tools were implemented more frequently for team-based care interventions for hypertension (62.5% and 56.3%) and diabetes (68.8% and 62.5%) than for other types of interventions. The use of a case manager/coordinator interaction was implemented more often for team-based care for diabetes (40.6%), referral to self-management programs for diabetes (37.5%), and self-management plan for hypertension (37.5%) than for other types of interventions (Table 2).

**Table 2:** Percentages of FQHCs Indicating Use of P/S/Ps, by Evidence-based Intervention and P/S/P Category, Illinois, 2016

Evidence-based Intervention	P/S/P Category			
	Internal Programs/Workflows, % (95% CI)	Huddles^a^, % (95% CI)	EHR^a^ Alerts/Tracking Tools, % (95% CI)	Case Manager/Coordinator, % (95% CI)
**Referral to self-management programs**				
Hypertension	81.2 (66.9–95.5)	43.8 (25.6–61.9)	34.4 (17.0–51.8)	21.9 (6.7–37.0)
Diabetes	80.6 (65.9–95.4)	59.4 (41.4–77.4)	34.4 (17.0–51.8)	37.5 (19.8–55.2)
**Team-based care**				
Hypertension	59.4 (41.4–77.4)	62.5 (44.8–80.2)	56.3 (38.1–74.4)	28.1 (11.7–44.6)
Diabetes	75.0 (59.1–90.7)	68.8 (51.8–85.7)	62.5 (44.8–80.2)	40.6 (22.6–58.6)
**Blood pressure self-monitoring**				
Hypertension	34.4 (17.0–51.8)	40.6 (22.6–58.6)	40.6 (22.6–58.6)	6.3 (0–15.1)
**Self-management plan**				
Hypertension	43.7 (25.6–61.9)	21.9 (6.7–37.0)	50.0 (31.7–68.3)	37.5 (19.8–55.2)

Abbreviations: CI, confidence interval; EHR, electronic health record; FQHC, Federally Qualified Health Center; P/S/Ps, policies, systems, and processes.

a Defined as 10-minute or less stand-up meetings used to foster communication in a clinical setting (11).

For controlled blood pressure, there was a significant difference (*P* < .05) in meeting or exceeding the national mean clinical QI percent score among FQHCs that implemented 9 or more P/S/Ps. Within each intervention category, there was a positive association between the percentage of FQHCs that had 3 or more P/S/Ps in place and met or exceeded the national mean clinical QI percent score for all hypertension and diabetes interventions. However, none were significant (Table 3).

**Table 3:** Overall and Within Interventions at or Below the Mean National Uniform Data System Clinical Quality Improvement Percent Score for Controlled High Blood Pressure and Poor Glycemic Control, Illinois, 2016

All P/S/P Categories^a^ (Combined)	% Did Not Meet	% Met or Exceeded	*P* Value^b^
**No. with controlled blood pressure^c^^,^ d**			
0–8	71.4	36.8	.049
9–16	28.6	63.2	
**No. with poor glycemic control (hemoglobin A1c >9%)^d^^,^ e**			
0–4	68.8	64.7	.81
5–8	31.2	35.3	
**Interventions (3 or more P/S/Ps)^f^**			
Referral (blood pressure)	28.6	42.1	.49
Team-based care (blood pressure)	35.7	63.2	.12
Self-monitoring (blood pressure)	7.1	21.1	.37
Self-management plan (blood pressure)	7.1	36.8	.10
Referral (diabetes)	37.5	41.2	.83
Team-based care (diabetes)	37.5	64.7	.12

Abbreviation: P/S/P, policies, systems, and processes.

a P/S/P categories were internal policies/workflows, huddles (10-minute or less stand-up meetings used to foster communication in a clinical setting [11]), electronic health record alerts/tracking tools, and case manager/coordinator interaction.

b *P* < .05 was considered significant.

c National mean Health Resources and Services Administration Uniform Data System score (2016) for controlled blood pressure, was 62.4% (14).

d χ^2^ test.

e National mean Health Resources and Services Administration Uniform Data System score (2016) for hemoglobin A_1c_ >9% was 32.1% (14).

f Fisher exact test was used to test level of significance when sample size was <5.

## Discussion

FQHCs achieve higher clinical QI percent scores for their patients with diabetes and hypertension compared with the national average (17). However, variation exists around what types and how many P/S/Ps are used to implement those evidence-based interventions among Illinois FQHCs. The FQHCs that responded to the IL-HISS indicated a high level of commitment to quality based on positive responses around Patient Centered Medical Home accreditation, meaningful use, use of supplemental QI software, and other attributes. In addition, our study showed that there was a positive association in the number of interventions implemented and mean clinical QI percent score for controlled hypertension (linear relationship) and poor glycemic control (inverse relationship).

National policy changes, such as Medicaid expansion, could have had an impact on the UDS clinical QI percent scores for FQHCs in Illinois (10). One study found that at federally funded community health centers, Medicaid expansion was associated with improved clinical quality for 4 of the 8 measures examined: asthma treatment, Papanicolaou testing, body mass index assessment, and controlled blood pressure (18). Additionally, our study did not account for patient-specific demographics and other factors like medication adherence, encounter frequency, and health literacy that affect chronic disease management (19–21). IL-HISS data are based on self-reported data. The study was cross-sectional and cannot conclude that the improved scores were due to the number or types of P/S/Ps implemented, only the association with them. Lastly, there is a possibility of nonresponse bias as a result of the methods used to collect these data.

There is growing evidence that use of P/S/Ps across health systems to improve the quality of chronic care is effective and vital to improving health outcomes (22). Our study focused on a subset of elements that address systematic supports around the implementation of evidence-based interventions that affect clinical QI percent scores. It may be useful to the public health community to study these elements in more detail. These could include availability of community resources, the level of leadership and decision support, and systems of care design to further assess interactions between health systems, care teams, and patients (23). The information gathered for our study will be used to improve training and technical assistance for FQHCs; specifically, in areas where gaps were identified (eg, increase the use of evidence-based interventions for blood pressure self-monitoring and self-management plans). Although the results of our study are not generalizable across states, they might be valuable for other states, territories, and local health jurisdictions to replicate this type of analysis to inform effective mechanisms (ie, types of P/S/Ps) that can be used to implement and sustain evidence-based interventions for hypertension and diabetes.

Initial results support some relationship between the number of P/S/Ps implemented and clinical QI percent score for both hypertension and diabetes. Given constraints in resources, staff time, and organizational infrastructure to support new or enhanced QI efforts, health systems would benefit from knowing what P/S/Ps work best for their organization. As the narrative around health care management and quality evolves, so too should the understanding around what composites of P/S/Ps have the greatest impact on health care outcomes for hypertension and diabetes.

## Appendix 2017 Illinois Health Information Systems Survey

Organizational Information:

1. Organization Name and Number of Sites
2. Current EHR system used within your organization and certification status
3. Does your organization use supplemental EHR software to support quality improvement and reporting efforts (eg, i2i, Azara, Mediquire, etc)?
4. What is the current status of Medical Home recognition within your organization?

Quality Reporting and Meaningful Use:

1. Has your organization reported on the National Quality Forum (NQF) Measure 0018 / Physician Quality Reporting System (PQRS) 236 in the past 12 months?
2. Has your organization reported on the NQF Measure 0059 in the past 12 months?
3. Does your organization use an EHR appropriate for treating patients (adults ≥18 years old) with high BP and/or diabetes?
4. Indicate the current stage (1, 2, or 3) of meaningful use and existing barriers to achieving meaningful use.

Electronic Health Record (EHR) Capabilities:

1. Does your EHR have the capability to generate a list of:
  - Adult patients (≥18 years old) with high BP and their BP reading for the past 12 months?
  - Adult patients (≥18 years old) that have high BP AND documentation of a self-management plan?
  - Adult patients (≥18 years old) diagnosed with diabetes?
  - Adult patients (≥18 years old) with an elevated blood glucose reading that are either undiagnosed as diabetic or that need follow-up?
  - Adult patients (≥18 years old) diagnosed with diabetes with a HbA_1c_ reading >9%?

Policies, Systems, and Processes:

1. What current policies or systems are used to refer adult patients (≥ 18 years old) with high BP to self-management resources? Are referrals to self-management resources being tracked?
2. What current policies or systems are used to encourage a team-based care approach to high BP control?
3. What policies or systems are in place to encourage BP self-monitoring?
4. What are the policies or systems in place to encourage healthcare providers to prescribe a self-management plan to adult patients (≥18 years old) with high BP and record the plan in the EHR?
5. Describe the system and/or process to tracking referral of patients with diabetes to self-management programs in the community.
6. What existing policies or systems are in place to encourage a team-based care approach to HbA_1c_ control?
7. What existing policies or systems are in place to encourage referral of persons with prediabetes or at high risk for type 2 diabetes to a CDC-recognized lifestyle change program?
8. Describe the system and/or process to tracking referral of persons with prediabetes or at high risk for type 2 diabetes to a CDC-recognized lifestyle change program.

## References

[1] National Center for Health Statistics. Health, United States, 2016: with chartbook on long-term trends in health. 2017. https://www.cdc.gov/nchs/data/hus/hus16.pdf. Accessed February 23, 2018. 28910066

[2] Benjamin EJ , Virani SS , Callaway CW , Chamberlain AM , Chang AR , Cheng S , Heart disease and stroke statistics — 2018 update: a report from the American Heart Association. Circulation 2018;137(12):e67–492. Erratum in: Circulation 2018;137(12):e493. 10.1161/CIR.0000000000000558 29386200

[3] American Diabetes Association. Standards of medical care in diabetes — 2014. Diabetes Care 2014;37(Suppl 1):S14–80. 10.2337/dc14-S014 24357209

[4] Chamberlain JJ , Rhinehart AS , Shaefer CF Jr , Neuman A . Diagnosis and management of diabetes: synopsis of the 2016 American Diabetes Association standards of medical care in diabetes. Ann Intern Med 2016;164(8):542–52. 10.7326/M15-3016 26928912

[5] Centers for Disease Control and Prevention. What is population health? https://www.cdc.gov/pophealthtraining/whatis.html. Accessed June 7, 2019.

[6] Institute of Medicine. Implementing a population-based policy and systems approach to the prevention and control of hypertension. In: Institute of Medicine. A population-based policy and systems change approach to prevent and control hypertension. Washington (DC): The National Academies Press; 2010. pp. 176–220.

[7] Graham G . Disparities in cardiovascular disease risk in the United States. Curr Cardiol Rev 2015;11(3):238–45. 10.2174/1573403X11666141122220003 25418513 PMC4558355

[8] Centers for Disease Control and Prevention. National diabetes fact sheet: national estimates and general information on diabetes and prediabetes in the United States, 2011. Atlanta (GA): US Department of Health and Human Services, Centers for Disease Control and Prevention; 2011.

[9] Agardh E , Allebeck P , Hallqvist J , Moradi T , Sidorchuk A . Type 2 diabetes incidence and socio-economic position: a systematic review and meta-analysis. Int J Epidemiol 2011;40(3):804–18. 10.1093/ije/dyr029 21335614

[10] Rothkopf J , Brookler K , Wadhwa S , Sajovetz M . Medicaid patients seen at federally qualified health centers use hospital services less than those seen by private providers. Health Aff (Millwood) 2011;30(7):1335–42. 10.1377/hlthaff.2011.0066 21734208

[11] Institute for Healthcare Improvement. Huddles. http://www.ihi.org/resources/Pages/Tools/Huddles.aspx. Accessed June 7, 2019.

[12] Health Resources and Services Administration. Uniform Data System. Reporting instructions for 2016 health center data. 2016. https://bphc.hrsa.gov/sites/default/files/bphc/datareporting/reporting/2016udsreportingmanual.pdf . Accessed January 24, 2019.

[13] Office of Disease Prevention and Health Promotion. Healthy people. https://www.healthypeople.gov/. Accessed September 12, 2018.

[14] Health Resources and Services Administration. Health center data and reporting. Health center program grantee data, 2016. https://bphc.hrsa.gov/uds2016/datacenter.aspx?year=2015. Accessed April 2, 2018.

[15] Office of Disease Prevention and Health Promotion. Heart disease and stroke. https://www.healthypeople.gov/2020/topics-objectives/topic/heart-disease-and-stroke/objectives. Accessed September 12, 2018.

[16] Office of Disease Prevention and Health Promotion. Diabetes. https://www.healthypeople.gov/2020/topics-objectives/topic/diabetes/objectives. Accessed September 12, 2018.

[17] National Association of Community Health Centers. Community health center chartbook, June 2017. http://www.nachc.org/wp-content/uploads/2017/06/Chartbook2017.pdf. Accessed November 6, 2017.

[18] Cole MB , Galárraga O , Wilson IB , Wright B , Trivedi AN . At federally funded health centers, Medicaid expansion was associated with improved quality of care. Health Aff (Millwood) 2017;36(1):40–8. 10.1377/hlthaff.2016.0804 28069845

[19] Neiman AB , Ruppar T , Ho M , Garber L , Weidle PJ , Hong Y , CDC grand rounds: improving medication adherence for chronic disease management — innovations and opportunities. MMWR Morb Mortal Wkly Rep 2017;66(45):1248–51. 10.15585/mmwr.mm6645a2 29145353 PMC5726246

[20] Morrison F , Shubina M , Turchin A . Encounter frequency and serum glucose level, blood pressure, and cholesterol level control in patients with diabetes mellitus. Arch Intern Med 2011;171(17):1542–50. 10.1001/archinternmed.2011.400 21949161 PMC3692291

[21] McNaughton CD , Kripalani S , Cawthon C , Mion LC , Wallston KA , Roumie CL . Association of health literacy with elevated blood pressure: a cohort study of hospitalized patients. Med Care 2014;52(4):346–53. 10.1097/MLR.0000000000000101 24556896 PMC4031281

[22] Wagner EH , Austin BT , Davis C , Hindmarsh M , Schaefer J , Bonomi A . Improving chronic illness care: translating evidence into action. Health Aff (Millwood) 2001;20(6):64–78. 10.1377/hlthaff.20.6.64 11816692

[23] The Commonwealth Fund. 2018 Scorecard on state health system performance. May 2018. https://www.commonwealthfund.org/publications/fund-reports/2018/may/2018-scorecard-state-health-system-performance. Accessed January 22, 2019.

